# Bringing leaders together to support the development of children’s palliative care across the Asia Pacific region—an interdisciplinary workshop

**DOI:** 10.3332/ecancer.2026.2149

**Published:** 2026-06-17

**Authors:** Gemma E Aburn, Rima Saad Rassam, Ximena Garcia-Quintero, Marta Salek, Andrea Cuviello, Yadurshini Raveendran, Sri Andini Handayani, Chen Chen Sun, Su Yadana, Sally Blair, Megan Doherty, Min Sun Kim, Zhou Xuan, Marianne B Phillips, Lee Ai Chong, Gayatri Palat, Donna Drew, Justin N Baker, Poh Heng Chong, Julia Downing, Michael J McNeil

**Affiliations:** 1 School of Nursing, University of Auckland, Auckland 1023, New Zealand; 2 Children’s Community Nursing, Waitemāta District, Te Whatu Ora Health New Zealand, Auckland 0610, New Zealand; 3 Department of Global Pediatric Medicine, St. Jude Children’s Research Hospital, Memphis, TN 38105, USA; 4 Division of Palliative Medicine, Phoenix Children’s Hospital, Phoenix, AZ 85016, USA; 5 Faculty of Medicine, University of Ottawa, Ottawa, ON K1H 8M5, Canada; 6 Two World’s Cancer Collaboration, North Vancouver, BC V7H 2Y8, Canada; 7 Department of Pediatrics, Seoul National University Hospital, Seoul 03080, Republic of Korea; 8 Haematology Center, Beijing Children’s Hospital; National Centre for Children’s Health, Capital Medical University, Beijing 100005, China; 9 Perth Children’s Hospital, Perth 6000, Australia; 10 International Society of Paediatric Oncology (SIOP) Oceania Palliative Care Working Group; 11 Pain and Palliative Care Unit, Universiti Malaya, Kuala Lumpur 50603, Malaysia; 12 Department of Pain and Palliative Medicine, MNJ Institute of Oncology and RCC, Hyderabad 500004, India; 13 Kids Cancer Centre, Sydney Children’s Hospital, Randwick, New South Wales 2000, Australia; 14 Division of Quality of Life and Pediatric Palliative Care, A Stanford School of Medicine and Stanford Medicine Children’s Health, Palo Alto, CA 94305, USA; 15 HCA Hospice Limited, 575348, Singapore; 16 International Children’s Palliative Care Network, UK; 17 Division of Quality of Life and Palliative Care, St. Jude Children’s Research Hospital, Memphis, TN 38105, USA; a https://orcid.org/0000-0002-9290-145X; b https://orcid.org/0000-0002-8232-8733; c https://orcid.org/0000-0003-1416-544X; d https://orcid.org/0000-0001-5570-3123; e https://orcid.org/0000-0002-7684-6841; f https://orcid.org/0000-0002-0523-0097; g https://orcid.org/0000-0003-2908-1329; h https://orcid.org/0009-0007-2207-8932; i https://orcid.org/0000-0003-0447-2083; j https://orcid.org/0009-0008-4423-725X; k https://orcid.org/0000-0003-3905-2169; l https://orcid.org/0000-0001-5323-9857; m https://orcid.org/0009-0000-6459-415X; n https://orcid.org/0000-0002-1963-4543; o https://orcid.org/0000-0001-7649-1449; p https://orcid.org/0000-0001-8803-0172; q https://orcid.org/0000-0002-5858-5131; r https://orcid.org/0000-0002-6584-6483; s https://orcid.org/0000-0003-4241-3295; t https://orcid.org/0000-0002-3450-785X; u https://orcid.org/0000-0001-8817-1995

**Keywords:** palliative care, paediatrics, quality of life, developing countries, capacity building, pain, interdisciplinary health team

## Abstract

Globally, there are over 21 million children in need of palliative care. Over a quarter (28%) of these children live in the Asia Pacific region, where access to children’s palliative care is limited or absent. Diversity in culture, economies, and political systems across the region has resulted in significant variation in how health services are developed, funded, and delivered. For children’s palliative care, services are often poorly coordinated across health systems, with limited integration into national health policies. Despite this there is a growing group of passionate clinicians and foundations trying to establish and develop services. This paper describes a 2-day leadership workshop that was held preceding the International Children’s Palliative Care Network conference in the Philippines in November 2025. The primary goal of the workshop was to introduce a health systems approach to equip children’s palliative care leaders and champions to advance palliative care development in their countries, at community, institutional, national and regional levels. The workshop brought together 51 children’s palliative care leaders, representing 17 countries/regions and 42 institutions across the region in addition to 28 international expert facilitators. Feedback from participants highlighted the value of this workshop for growth and development across policy, research, clinical service development, education and training. This workshop lays the foundation for further collaboration and partnerships across Asia Pacific and the world to strengthen children’s palliative care.

## Introduction

Children’s palliative care is comprehensive and compassionate care that addresses the physical, psychosocial, spiritual and cultural needs of infants, children and young people with serious illness and their families. To optimise quality of life for children and their families, palliative care should be available to every child facing a life-threatening, life-limiting, or serious condition [1–3]. Access to children’s palliative care remains a significant challenge for children living in low- and middle-income countries (LMICs). Globally, there are over 21 million children requiring palliative care [4] with 98% of children with serious health-related suffering living in LMICs.

Almost 28% of children in need of palliative care reside in Asia Pacific. While well-established palliative care for children exists in some high-income countries within Asia Pacific, most countries in the region have limited or no established children’s palliative care services [4, 5]. For the purposes of this work, the Asia Pacific region refers to a geographical area encompassing all areas of Asia, Oceania and the Western Pacific. Asia Pacific’s diverse cultures, economies, and political systems lead to difference in how health services are developed, funded, and delivered. For children’s palliative care, services are often poorly coordinated across health systems and lack integration into national health policies [6]. Moreover, inadequate access to essential medicines and a shortage of trained health professionals - both generalists and specialists in children’s palliative care remain frequent barriers [7].

Over recent years, the Asia Pacific Hospice and Palliative Care Network (APHN), has focused on catalysing the development of children’s palliative care across the region, through regional education and training initiatives [8]. By sharing lessons learned and nurturing a regional special interest group (SIG), APHN has facilitated knowledge exchange and accelerated the adoption of effective strategies for palliative care development in the region. The SIG also provides a platform for collaboration on challenges related to clinical service development, policy, and research [8, 9].

In November 2025, a global children’s palliative care conference was hosted by the International Children’s Palliative Care Network (ICPCN) in Manila, Philippines, with a 2-day pre-conference workshop designed to strengthen leadership and collaboration across the region. We report the workshop’s design, implementation and future implications for children’s palliative care in the region. This paper refers to children’s palliative care, this encompasses the care of neonates, children and adolescents with a life-limiting or life-threatening condition.

## Design of the workshop

### Purpose and goals

The workshop goal was to introduce a health systems approach to equip children’s palliative care leaders and champions to advance palliative care development in their countries: at community, institutional, national and regional levels. The workshop introduced participants to the World Health Assembly resolutions on Palliative Care [10] and Cancer [11, 12], the most recent mapping of national palliative care development in the Asia Pacific region [7] and the ICPCN global mapping of children’s palliative care [13] as key tools which can be used to support action on palliative care.

### Design, sample and setting

The 2-day Children’s Palliative Care Leadership Workshop for Asia Pacific was held prior to the ICPCN conference. The workshop was co- organised by St. Jude Children’s Research Hospital in partnership with the American Lebanese Syrian Associated Charities (ALSAC), ICPCN and the Ruth Foundation, in collaboration with APHN, Stanford Medicine Children’s Health, and Education in Palliative and End of Life Care – Pediatrics (EPEC-Peds). This interprofessional workshop used the World Health Organisation (WHO) conceptual model for palliative care development [14] to consider how children’s palliative care could be progressed in the region across health workforce education and training, research, health policy, empowerment of communities, use of essential medicine and the provision of palliative care through integrated health services.

Workshop participants were identified through the St Jude Global Alliance and ICPCN and confirmed with the APHN Paediatric SIG leadership team and members of the organising committee. These organising institutions had exisiting relationships across the region and were able to ensure wide representation of interdisciplinary professionals, including health professionals from various disciplines, policy makers, non-governmental organisations, and charitable foundations. The workshop brought together 51 children’s palliative care leaders, representing 17 countries/regions and 42 institutions across the region in addition to 28 regional and global experts from Australia, Switzerland, South Africa, United Kingdom, and United States (Appendix 1). Demographics of participants are shown in Table 1. Participants represented diverse interprofessional disciplines, including medical doctors (55%; *n* = 28), nurses (16%; *n* = 8), social workers (4%; *n* = 2) and representatives from foundations and not for profit organisations (25%; *n* = 13).

## Delivery methods

The workshop was held in-person in November 2025, immediately preceding the ICPCN Conference. Regional and global leaders met online routinely beginning in June 2025, to plan workshop content and sessions. The workshop programme (Appendix 1) highlighted regional developments and best practices and provided opportunities for participants to use the WHO conceptual model for palliative care to co-design approaches that were context-specific and regionally aligned. The workshop content combined expert-led presentations, panel sessions, case studies and small-group activities paired with design thinking exercises to generate collaborative and novel solutions addressing each of the WHO framework pillars. Workshop content was adapted from a successful programme conducted in the Eastern Mediterranean region, in 2024 [15].

Over the last decade, the human-centered design approach has gained prominence in developing collaborative, user-centered healthcare solutions that address complex challenges [16]. This approach provided the foundation for continuous engagement throughout the 2-day event. Another factor that bolstered continued dialogue was the ICPCN conference on the following days, which amplified interactions within the larger global community of children’s palliative care. The well-recognised EPEC-Pediatrics Professional Development workshop (PDW) [17] was also integrated into day 2 of the programme for a small subset of participants, who focused on the education and training pillar of the WHO conceptual framework [14].

## Workshop evaluation survey

At the end of the workshop, participants received a QR code linking to an electronic survey designed specifically for the workshop. The survey combined satisfaction ratings, learning evaluation, and reflections on personal development, using both five-point Likert scales and open-ended questions. This allowed workshop experience and the potential impact on advancing children’s palliative care across the region. Quantitative data were analysed using descriptive statistics, while open-ended responses underwent thematic analysis.

## Summary of the workshop

### Setting the scene

The workshop was opened with introductions from leaders of global, regional and local collaborators who organised the workshop. Presenters alluded to significant progress in service development, compared to before, but access to care still largely concentrated within urban areas and major hospitals. While passion in developing children’s palliative care is high across Asia-Pacific, maintaining the momentum for action remains a challenge between events and meetings. This workshop was seen as an opportunity to consider development within services and countries, but also to contemplate regional collaboration and connection moving forward.

To facilitate introductions of participants, the ALSAC team led an interactive session on developing your ‘elevator pitch’ [18]. Participants developed a 30-second pitch and practiced their pitches with peers. This provided an opportunity for shared energy in the room and willing engagement of participants in the programme moving forward.

The morning concluded with a presentation of the Asia-Pacific findings of the Assessing Doctors Attitudes on Palliative Treatment (ADAPT) study. ADAPT is an international study that has used a mixed methods survey to explore the perceptions of medical doctors, regardless of specialty, who care for children diagnosed with cancer toward palliative care integration [19]. This study highlighted barriers in integrating children’s palliative care into the care of children with cancer, including how children’s palliative care is perceived by clinicians [20–22]. This provided a useful platform to consider barriers and opportunities in small group work later in the workshop.

**Figure 1.**
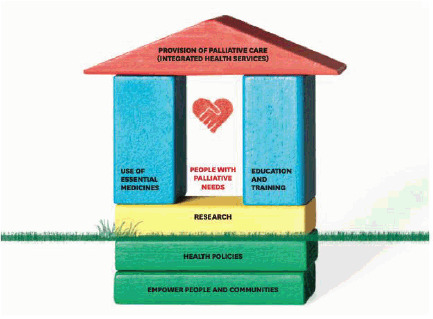
The WHO conceptual framework for palliative care development that underlies the design of the workshop design and activities based on six pillars [14].

Following an informal networking break, participants were introduced to the conceptual framework for palliative care development – Figure 1 [14]. They were also provided with their countries report from the recently published APHN Atlas of palliative care in the Asia Pacific Regions 2025 [7]. Participants then critiqued these reports and considered how they could build on these findings to develop children’s palliative care in their home countries.

## Group activities

Participants were divided into six small groups, each group focusing on a pillar of the conceptual framework shown in Figure 1: Empower People and Communities, Health Policies, Research, Use of Essential Medicines, Education and Training, and Provision of Palliative Care. Design thinking activities included:

- Cause/Effect or Fishbone diagram to focus on the specific problem within the pillar and delve into challenges
- Roses and buds to collectively explore the regional strengths and opportunities to initiate/leverage burgeoning efforts navigating the challenges within the pillar
- Random word exercise to probe creative thinking of solutions to promote the WHO pillar
- Impact-effort matrix to prioritize the pool of solutions based on evidence of effectiveness and required resources.
- Mapping process to co-design the steps for implementing the prioritized solution

Groups highlighted the core challenge and moved to identify one activity that could develop children’s palliative care within a specified time frame (Table 2).

**Figure 2.**
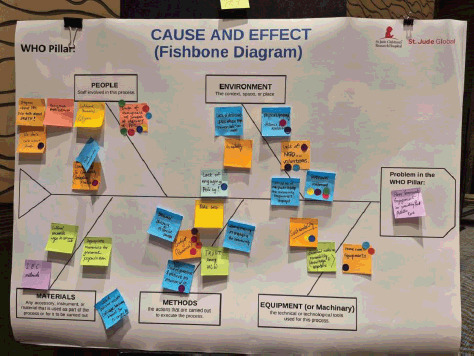
A sample of a cause-effect (Fishbone) diagram created by the group assigned to the WHO pillar on “Empowering People and Communities” during the workshop.

**Figure 3.**
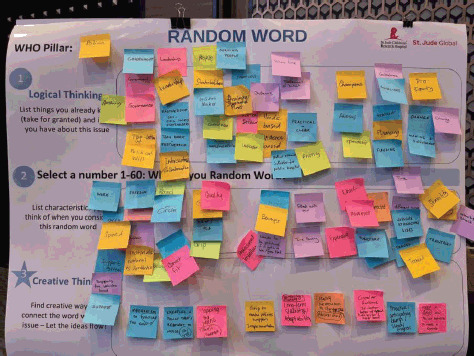
A sample of a random word activity by the group assigned to the WHO pillar on “Policies” during the workshop.

**Figure 4.**
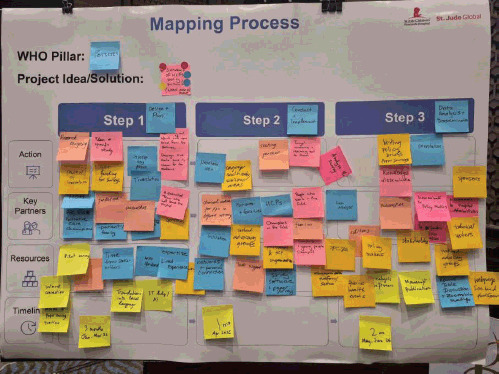
A sample of a process mapping activity by the group assigned to the WHO pillar on “Policies” during the workshop.

Examples of the process groups worked through are shown below in Figures 2–4.

## Regional panel sessions

Panel sessions were incorporated throughout the 2-day programme (Appendix 1), offering the opportunity to hear from regional leaders who shared their experiences in developing services, policies and advocacy initiatives in children’s palliative care. These sessions were enlightening and highlighted the progress achieved in the region to date.

One panel discussion showcased five success stories from India, China, Papua New Guinea, Indonesia and the Philippines. Key takeaway messages included: the importance of using data and stories to support advocacy, the urgency of developing clinical guidelines to strengthen care delivery, the need to empower communities to drive change and the critical role of awareness and education across community, health professionals, policy makers and government.

Another panel session explored the role of partnerships and the exchange of expertise between regional and global children’s palliative care advocates. Represented institutions were Indira Cancer Trust from Sri Lanka, Golden Butterflies Children’s Palliative Care Foundation from India, the Ruth Foundation from Philippines, St. Jude Children’s Research Hospital and the WHO. Panelists shared insights and recommendations on advocacy and partnership. Discussions emphasised respecting culture and community dynamics, with cultural, religious, and linguistic diversity identified as a key to fostering trust and acceptance of children’s palliative care. Moreover, engaging policymakers and diversifying partners including the WHO and multi-sector stakeholders, was highlighted as essential for aligning with global norms and building a compassionate network across the sector. Finally, panelists reflected on the need for transparent data documentation and a shared vision to ensure sustainability, build trust, advocate for children’s palliative care, and empower local leaders to drive systemic change. Finally, education was a central theme throughout the 2-day programme and featured prominently across all pillars. The group with a focus on education and training, was identified in advance as leaders in the education space and invited to participate in an EPEC-Pediatrics PDW.

## EPEC-Pediatrics PDW

The EPEC-Pediatrics PDW is the third stage in the EPEC-Pediatrics programme, following the end-user and train the trainer programmes. The PDW is intended for clinicians who wish to engage in teaching train the trainer programmes [15]. Holding this workshop as part of the leadership programme provided an opportunity to develop trainers and future capacity for running EPEC-Pediatrics programmes across the region. Thirteen participants attended this workshop, representing eight countries. Of the group, seven participants were medical doctors, five were nurses, and one participant was a leader of a foundation with lived experience of children’s palliative care. The participants were supported by an international EPEC-Pediatrics leader, and five experienced EPEC-Pediatrics senior lecturers from across the region. This was a unique experience integrating an EPEC-Pediatrics PDW into a leadership programme, and both facilitators and participants reflected on the opportunity to integrate broader considerations in children’s palliative care alongside the education focus.

## Resources

Before the closure of the workshop, some of the existing literature, educational materials, and networking resources to support planning and implementing children’s palliative care initiatives, were outlined. These resources provide practical guidance for advocacy and the delivery of high-quality children’s palliative care services.

## Participants’ perspectives

**Figure 5.**
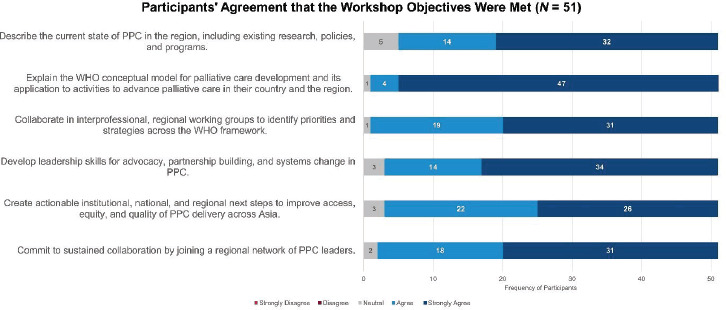
The frequency of participants’ responses on each level of agreement with meeting the objectives of the workshop. Out of the 51 participants, 46 “agreed” or “strongly agreed” that all the workshop objectives were met.

All participants completed the post-workshop electronic survey with a 100% response rate. This response rate perhaps represents the dedication and commitment of this group to development of children’s palliative care. At least 46 participants agreed or strongly agreed that the workshop objectives were met (95%–99% of respondents). This is shown in Figure 5 below.

For 90% (*n* = 46) of participants more than half of the workshop content was new. Additionally, 92% (*n* = 47) agreed or strongly agreed that the topics were relevant to practice and that the workshop struck an appropriate balance between theory and practical skills.

Participants evaluated **knowledge gained** from each session: the elevator pitch was rated most helpful (100%, *n* = 51 agreed or strongly agreed), followed by the WHO pillars fishbone diagram (98%, *n* = 50 agreed or strongly agreed).

**Figure 6.**
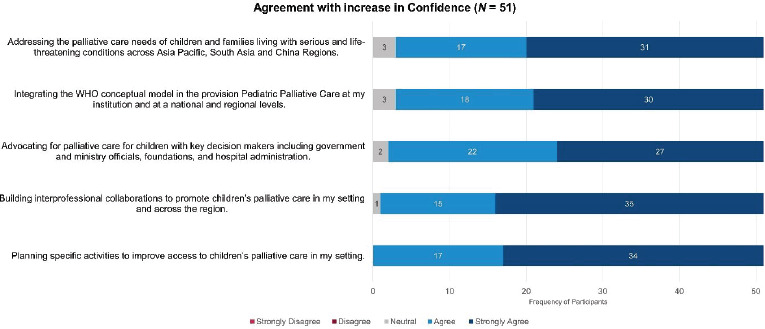
Participant feedback – agreement with increase in confidence. Figure 6 illustrates the frequency of participants’ responses on each level of agreement with statements that reflect increasing confidence in addressing children’s palliative care aspects covered in the workshop.

Confidence increased across multiple domains; 98%, *n* = 50 agreed or strongly agreed on the value of building interprofessional collaborations to advance children’s palliative care locally and regionally. Participants also gained confidence in planning specific activities to improve access to children’s palliative care (100% *n* = 51 agreed or strongly agreed). Other areas in which participants gained confidence are shown in Figure 6.

Overall, the workshop delivery was rated positively with all participants appreciating the organisation of the workshop, the networking opportunities to connect with participants and faculty leaders, workshop length and session timing as ‘good’, ‘very good’ or ‘excellent’. Feedback suggested that future workshops could benefit from allocating additional time for questions, with 4% of participants rating the opportunity to ask questions as ‘poor’.

Participants’ feedback demonstrated that the workshop was beneficial, with 98% (*n* = 50) of participants reporting that the workshop met their personal goals and objectives. Participants also responded to free-text questions outlining their intended next steps. Thematic analysis of these responses identified three core themes: learning and education; collaboration and connection; and implementation and application. Although the paper refers to Children’s Palliative Care, participants’ quotes will sometimes refer to Paediatric Palliative Care (PPC) – these have been left as written by participants.

### Learning & education

Participants highlighted the value of education and the need to develop curriculum and training to support clinicians in caring for children with palliative care needs and their families:

*“Create PPC Curriculum”*

*“Develop a training programme and pioneer PPC in [my own country]”*

*“EPEC training in [my own country]”*

Modes of teaching skills were also reflected in feedback, highlighting the value of teaching leaders’ different education techniques.

*“Design more role-play-based teaching”*

### Collaboration and connection

Collaboration and connection were highlighted in feedback, where participants valued the opportunity to *“network and connect with regional colleagues”*. In developing programmes and considering opportunities, participants also considered the need to *“co-operate with government”* and* “connect more with PPC advocates”.*

### Implementation and application

This theme alluded to the passion in the group for progressing children’s palliative care in the region. Participants highlighted several activities and strategies they wanted to implement following this programme. These activities included research, for example *“ADAPT in Australia and more Pacific Sites”,*

*“Improving PPC support in our foundation”,* empowering communities and several participants felt there was a need to *“conduct a similar workshop in our institution”.*

Overall participants shared overwhelmingly positive insights on the opportunity to attend this workshop as reflected in the below quote:

*“The workshop was well-organised, insightful, and inspiring. The diversity of participants and facilitators fostered meaningful exchange of ideas across the Asia-Pacific region. Sessions were practical and reflective, providing both strategic and emotional insights into leading palliative care programs. Travel coordination and logistics were smooth, and the supportive environment made learning and collaboration very effective”*

While there was very little that participants identified could be improved with the programme, a small number of participants commented that having hard copies of the presentations would be beneficial for their learning.

## Potential impact and next steps

While participant feedback reflected strong regional energy, at the workshop’s conclusion, it is important to consider how this momentum can be sustained. The workshop’s opening highlighted the recurrent challenge: enthusiasm at events often diminished over time. A debrief meeting has been held with global and regional organisers to reflect on the experience and ongoing impetus. Success is rooted in the shared goal of alleviating children’s suffering and a commitment to learning from and empowering one another to ensure equitable access to children’s palliative care. Although the workshop spanned over 2 days, it preceded the ICPCN conference, allowing participants to further enhance their networking experience. Regional and global meetings, similar to the ICPCN conference, provide valuable platforms for dialogue and collaboration. Ongoing mentorship and support from international experts and leaders was seen as essential. A WhatsApp group was established for the workshop, enabling real-time discussion during the workshop and conference. This group remains a potential forum for sharing success stories, challenges, peer support, resources, news, and upcoming events.

Several participants have already communicated plans for education and training workshops in 2026. For example, participants from Fiji and Tonga are preparing a comprehensive development programme and education roll out to health professionals and communities later this year; EPEC-Pediatrics is scheduled for April in Hong Kong; and there has been the development of a topic guide for small group teaching throughout 2026 in Papua New Guinea. Participants who have advanced next steps, all had pre-existing relationships with regional facilitators serving as mentors, underscoring the importance of ongoing mentorship and partnership, to ensure collective progress can be made across the region.

## Conclusion

This workshop marked an important milestone in advancing children’s palliative care across the Asia-Pacific region. It provided a forum for networking and collaboration within and between organisations, and a space to explore strategies to sustain momentum in improving care of children with serious illness locally, nationally and across the region. The WHO conceptual framework for palliative care development [14], served as a guide for progress to be considered across key areas: research, education and training, community empowerment, policy, access to essential medicines, and importantly the provision of palliative care to children with serious illness and their families. Through activities grounded in human-centred design principles, participants identified challenges, resources, and opportunities for development and change. Participant feedback demonstrated measurable benefits from the workshop, however, strategies to maintain momentum and to achieve long-term outcomes will require continued attention in future work.

## Conflicts of interest

Nil.

### Funding

This workshop was funded by the American Lebanese Syrian Associated Charities (ALSAC). There was no funding for the preparation of this manuscript.

### Author contributions

GEA and RSR wrote the manuscript. All authors engaged in the design of the report and reviewed and approved the manuscript.

## Reference

## Figures and Tables

**Figure:**
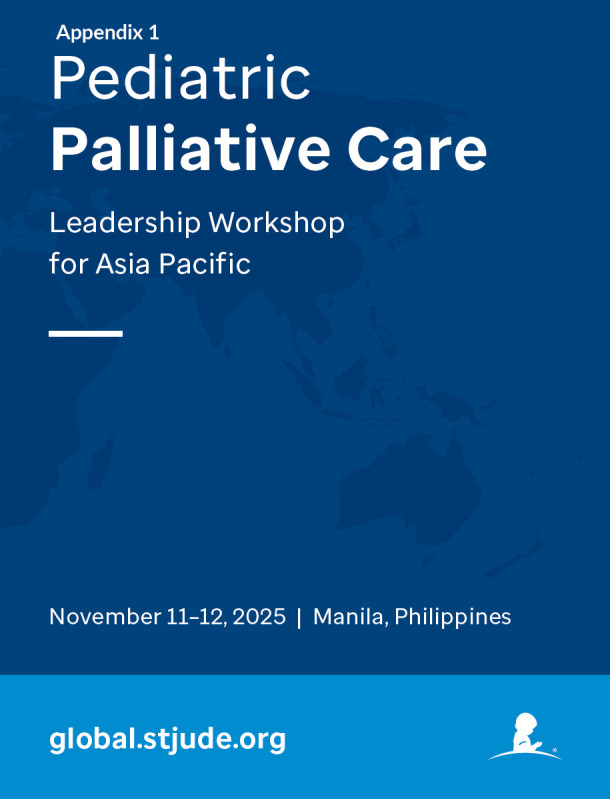


**Figure:**
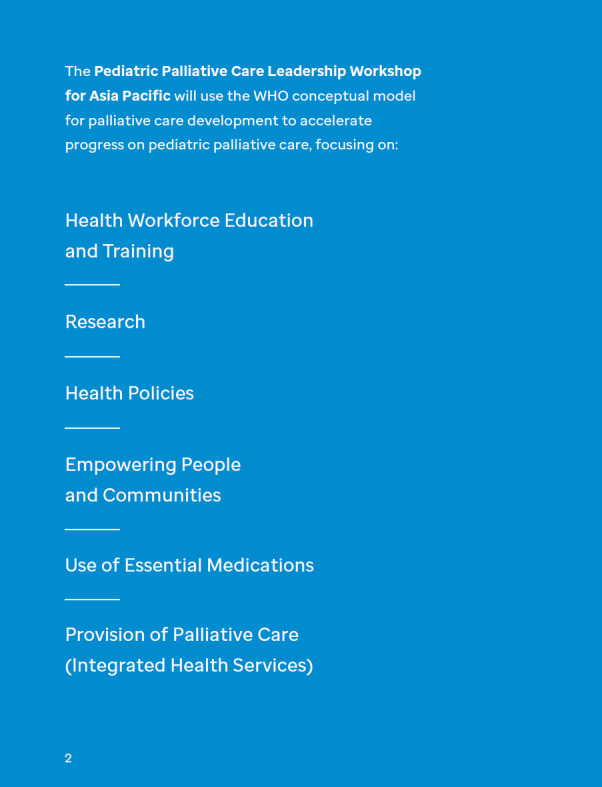


**Figure:**
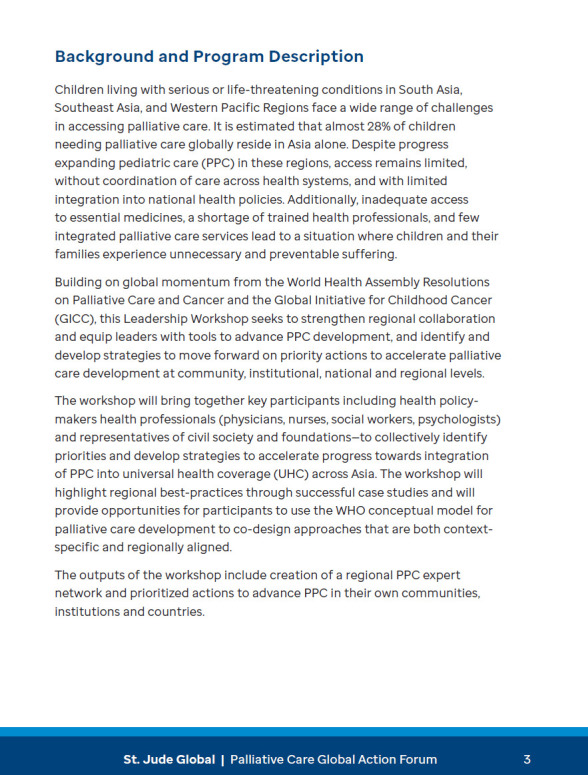


**Figure:**
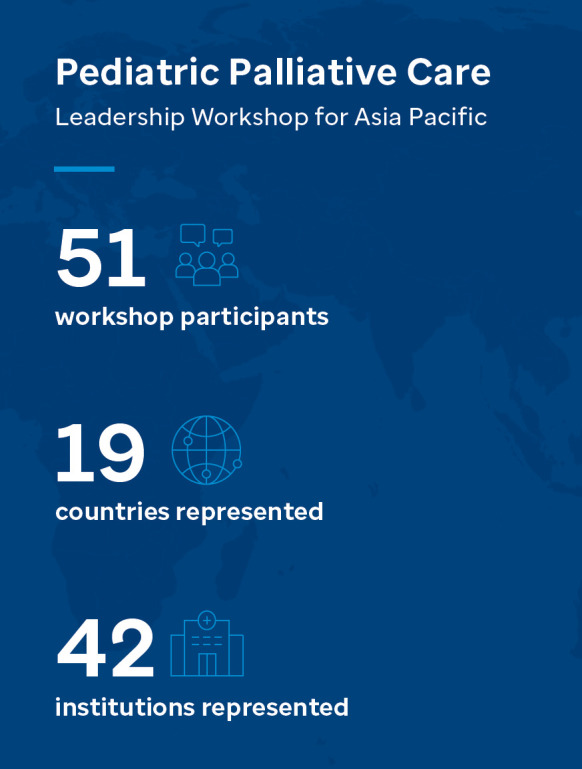


**Figure:**
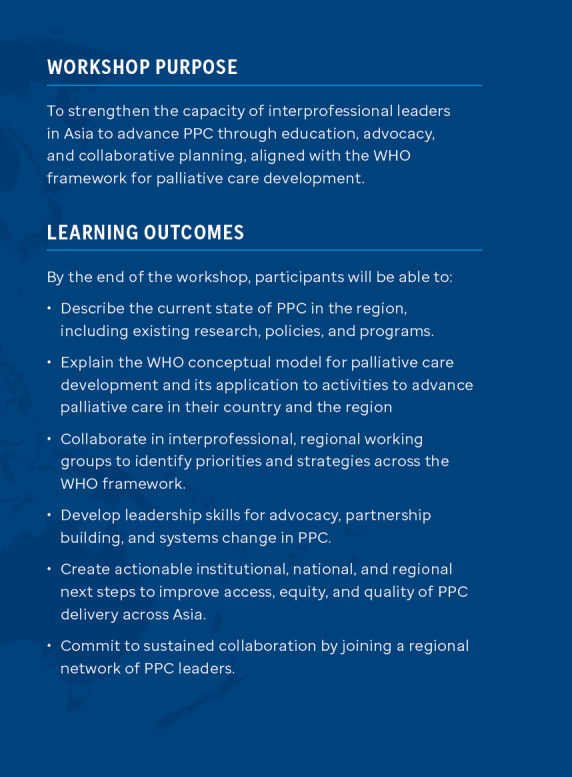


**Figure:**
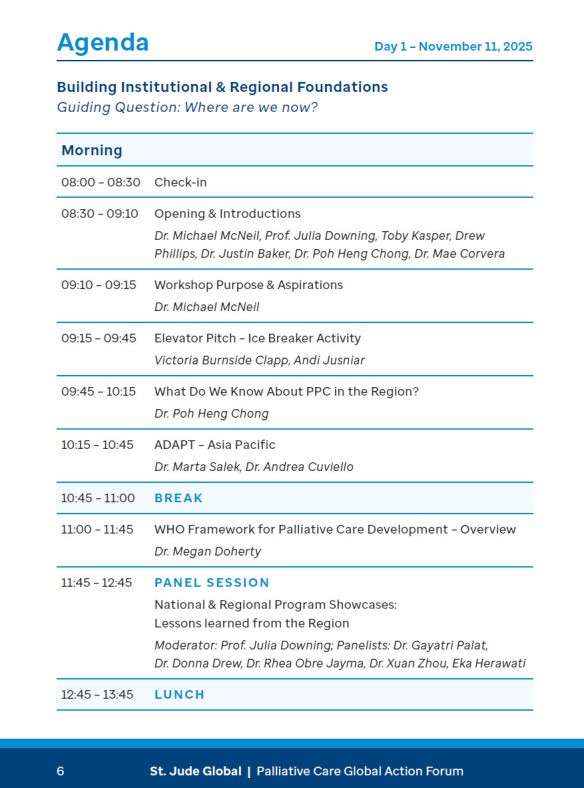


**Figure:**
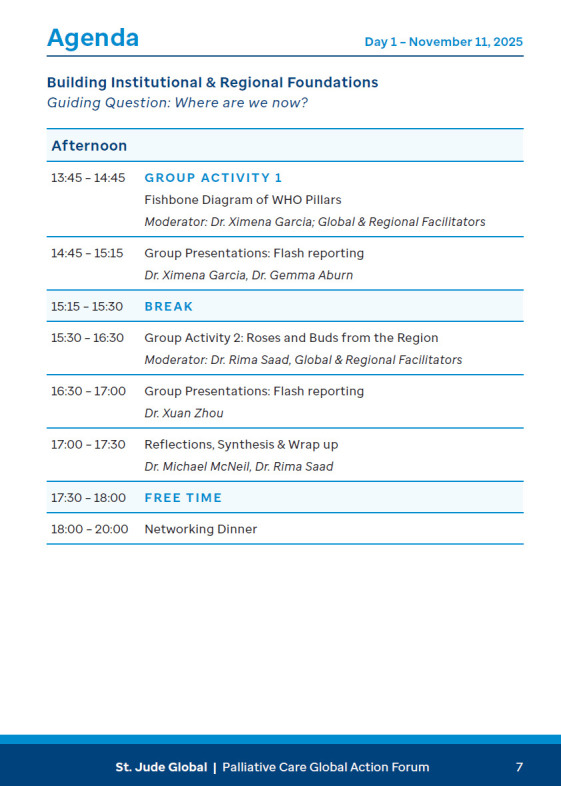


**Figure:**
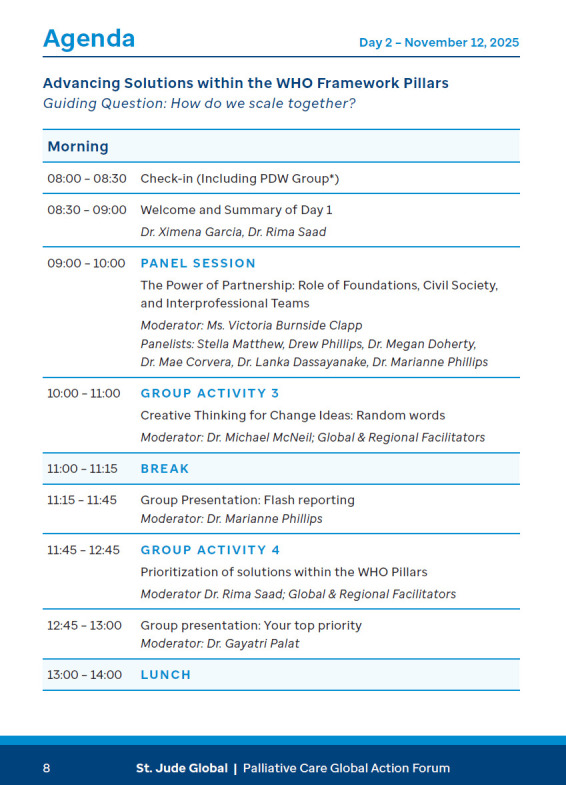


**Figure:**
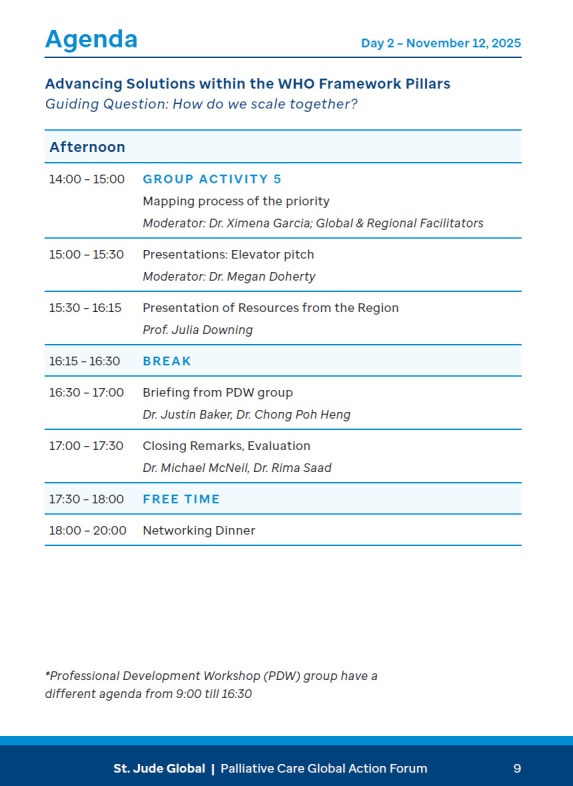


**Figure:**
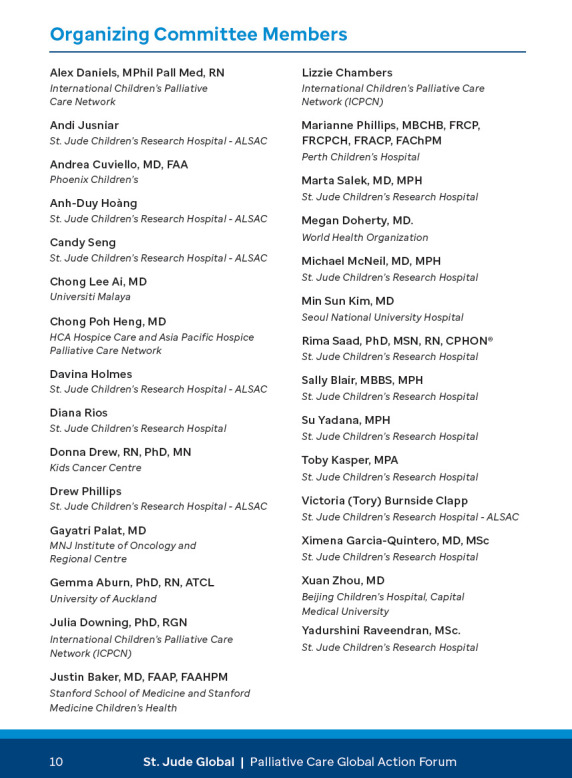


**Figure:**
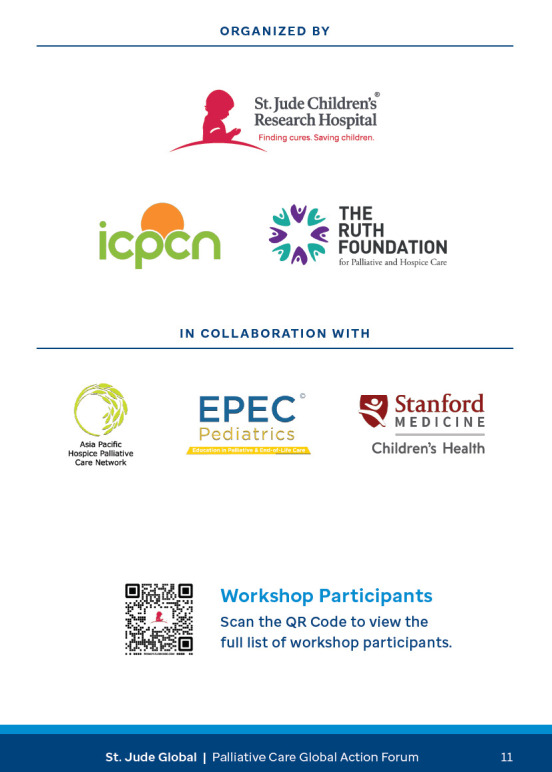


**Figure:**
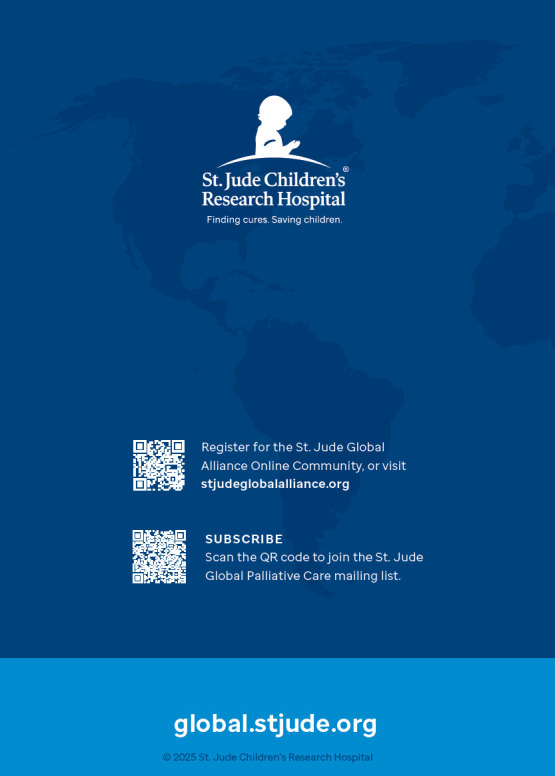


**Table 1.** Demographic characteristics of participants (n = 51).

Characteristic	Number (n)	Percentage (%)
Disciplines		
Medical doctor	28	55
Nurse	8	16
Social worker	2	4
Charitable foundation representative ^a^	13	25
Years of experience in the role		
< or = 1	1	2
2–5	17	33
6–10	19	37
11–15	4	8
> 15	10	20
Countries/Regions		
Philippines	16	31
Mainland China	6	12
India	5	10
Hong Kong China	3	6
Fiji	2	4
Indonesia	2	4
Malaysia	2	4
Papua New Guinea	2	4
South Korea	2	4
Singapore	2	4
Timor Leste	2	4
Vietnam	2	4
Nepal	1	2
New Zealand	1	2
Sri Lanka	1	2
Thailand	1	2
Tonga	1	2

Table 1 presents the number and percentage of each characteristic among participants with regards to distribution of disciplines, years of experience, and represented countries ^a^ Two participants have a clinical background as Nurse and Child Life Specialist in addition to being a Charitable Foundation Representative

**Table 2.** Identified challenges and activities to develop children’s palliative care.

WHO pillar	Problem highlighted	Prioritized solution and timeline
Empowering people & communities	Poor community engagement	Education & Training for Communities (March 2027)
Research	Lack of knowledge & resistance	Assess perceptions of health professionals about children’s palliative care research (August 2026)
Policy	Lack of implementation of policy	Survey of health professionals about policies – needs assessment (May-June 2026)
Education & Training	Lack of education programmes	EPEC-Pediatrics programmes planned across the region ongoing (next April 2026)
Use of essential medicines	Lack of access to WHO recognised essential medicines	Creation of advocacy group (July 2027)
Provision of palliative care	Inadequate and inequitable provision of children’s palliative care	Improve community awareness of children’s palliative care (Dec 2027)

Table 2 summarizes prioritized barriers to Children’s Palliative Care within the WHO pillars and the initial plans to mitigate each barrier in the Asia Pacific Region
